# Demonstration of Heterogeneous Structure for Fabricating a Comb-Drive Actuator for Cryogenic Applications

**DOI:** 10.3390/mi13081287

**Published:** 2022-08-11

**Authors:** Gaopeng Xue, Masaya Toda, Xinghui Li, Bing Li, Takahito Ono

**Affiliations:** 1 School of Mechanical Engineering and Automation, Harbin Institute of Technology, Shenzhen 518055, China; 2 Tsinghua Shenzhen International Graduate School, Tsinghua University, Shenzhen 518055, China; 3 Graduate School of Engineering, Tohoku University, Sendai 980-8579, Japan

**Keywords:** comb-drive actuator, heterogeneous structure, cryogenic application, displacement sensor

## Abstract

This study presents an experimental demonstration of the motion characteristics of a comb-drive actuator fabricated from heterogeneous structure and applied for cryogenic environments. Here, a silicon wafer is anodically bonded onto a glass substrate, which is considered to be a conventional heterogeneous structure and is commonly adopted for fabricating comb-drive actuators owing to the low-cost fabrication. The displacement sensor, also with comb-finger configuration, is utilized to monitor the motion characteristics in real time at low temperatures. The irregular motions, including displacement fluctuation and lateral sticking, are observed at specific low temperatures. This can be attributed to the different thermal expansion coefficients of two materials in the heterogeneous structure, further leading to structural deformation at low temperatures. The support spring in a comb-drive actuator is apt to be deformed because of suspended flexible structures, which affect the stiffness of the support spring and generate irregular yield behavior. The irregular yield behavior at low temperatures can be constrained by enhancing the stiffness of the support spring. Finally, we reveal that there are limited applications of the heterogeneous-structure-based comb-drive actuator in cryogenic environments, and simultaneously point out that the material substrate of silicon on the insulator is replaceable based on the homogeneous structure with a thin SiO_2_ layer.

## 1. Introduction

Precision positioning of microactuators has attracted great interests owing to their practical applications in diverse fields, such as scanning probe microscopy (SPM) [1,2], probe-based data-storage systems [3,4], and micro-optical systems [5,6]. Up to now, various types of microactuators have been well developed on the basis of the actuation mechanisms of electrostatic [7,8], piezoelectric [9], thermoelectric [10], and electromagnetic [11]. Among these, electrostatic-based microactuators have been extensively studied as attractive positioning devices because of their large displacement range, easy manufacturing, and high system integration [12,13,14,15]. In particular, comb-drive actuators consisting of two interdigitated finger structures have been widely employed as microgrippers [16,17], optical shutters [18], micromechanical gears [19], and multidimensional microstages [20,21,22,23,24].

Here, multidimensional microstages, applied for SPM measurement systems, require large strokes along multiple directions with nanometer-level positioning accuracy. Recently, a burgeoning magnetic resonance force microscopy (MRFM) technique, combined with atomic force microscopy detection mechanism, can non-invasively detect the densities of radicals by using a magnet-mounted cantilever [25,26,27,28]. Usually, a cryogenic environment for measuring the magnetic resonant force is required to reduce the thermomechanical noise and improve the cantilever-resonance sensitivity [29,30,31]. Thus, microscanners with the ability of offering large strokes at low temperatures is more attractive for MRFM cryogenic measurements. Notably, the conventionally piezoelectric-actuation-based microstages provide attenuated displacement strokes with a decrease in temperature, which extremely constrains the applications for 3D scanning of large-scale species such as cells [32]. In contrast, the comb-drive actuator is a suitable candidate since the actuation mechanism is not affected by temperature, indicating that relatively large displacements can be obtained at low temperatures [33].

In terms of the comb-drive actuator applied for low temperature environments, except the actuation mechanism, the structural design as well as the material substrate should also be considered for fabricating the functional structures. On the basis of microelectromechanical systems (MEMS) microfabrication technology, silicon-on-insulator (SOI) is the most frequently used material substrate for fabricating various MEMS devices, such as comb-drive actuators. However, as compared with the expensive cost of SOI wafer, a conventional heterogeneous structure of silicon (Si) wafer bonded onto glass substrate is an alternative material substrate owing to its low-cost fabrication. Until now, the feasibility of a heterogeneous-structure-based comb-drive actuator for cryogenic applications has been still unverified. In addition, the support spring in a comb-drive actuator, suspends the movable comb fingers and play an integral role in constraining the lateral instability and achieving large displacements [34]. Hence, the structural stiffness of the support springs depend on the temperature, which needs to be explored for specifically analyzing the motion characteristics at low temperatures.

In this study, we experimentally demonstrated the feasibility of using a heterogeneous structure for fabricating a comb-drive actuator for cryogenic applications. A conventional heterogeneous structure consisting of Si bonded onto glass is adopted as the substrate material because of the low cost and simple fabrication. Two types of support springs with different stiffnesses are designed in comb-drive actuators for validating the temperature affection on structural stiffness and further analyzing the motion characteristics at low temperatures. A displacement sensor, also with comb-finger configuration, is embedded into the comb-drive actuator, and is able to monitor capacitance changes in real time and further reflect the motion situation. Finally, the heterogeneous-structure-based comb-drive actuators are fabricated based on the microfabrication technology, and then loaded into the vacuum chamber of a cryostat for characterizing the actuation performance.

The remainder of this paper is organized as follows: In Section 2, we present the structural design and parameters of the comb-drive actuator; in Section 3, we describe the microfabrication process and experimental results of the devices; in Section 4, we conduct the cryogenic experiments of the fabricated comb-drive actuators; in Section 5, we summarize our findings and proposes our future work.

## 2. Design

**Figure 1:**
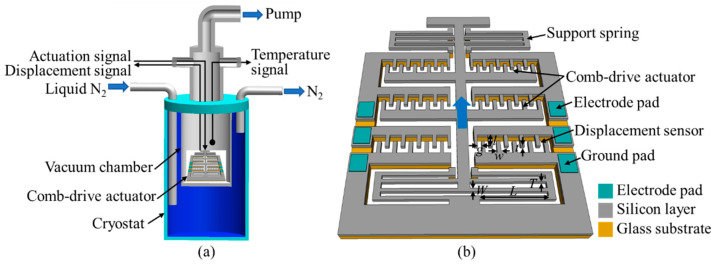
(**a**) Schematic of cryogenic setup for measuring the comb-drive actuator at low temperatures; (**b**) schematic of the one-axis comb-drive actuator fabricated from the heterogeneous structure.

Figure 1a shows a cutaway view of the cryogenic setup for measuring the actuation performance of the comb-drive actuator at low temperatures. The comb-drive actuator is wire-bonded onto a printed board, and then located into a vacuum chamber of the cryostat. Prior to importing the liquid nitrogen (N_2_) into the cryostat to cool down the microstage, the vacuum chamber is completely pumped by a molecular pump connecting with a rotary pump. A temperature sensor is utilized to monitor the temperature information of the vacuum chamber in real time. The actuation voltages from the external power sources are applied to the comb-drive actuator, and the corresponding displacement signal from the displacement sensor of the comb-drive actuator is detected by the external sensing circuit. Thus, the actuation performance of the comb-drive actuator at low temperatures can be obtained by analyzing the output displacement signals. Figure 1b depicts the schematic of the designed one-axis comb-drive actuator, which is fabricated from a heterogeneous structure, which consists of a Si wafer anodically bonded onto glass substrate as our experimental target. All the functional structures of the comb-drive actuator are designed onto the Si wafer, whereas the glass substrate serves as the anchor supporting the fixed and movable interdigital comb-finger electrodes. In the Si wafer layer, several rows of the interdigital comb-finger electrodes, acting as the actuation units, are distributed in the center region for providing one-axis displacement stroke. The displacement sensor, also based on the comb-finger layout, is designed next to the actuation units for precisely sensing the actuated displacements. Two support springs with folded-flexure layout, located in the upper and lower positions of the actuation units, support the movable comb-finger parts and offer the restoring force to generate the desired displacement. Because the vacuum chamber of the cryostat seems to be a black box, i.e., no optically characterizing function, the actuation performance of the comb-drive actuator at low temperatures can only be monitored in real time by utilizing a displacement sensor. By comparing the displacement signals between room temperature and low temperature, we can obtain the motion characteristics of the comb-drive actuator at low temperature.

To analyze the influence of low temperature on the motion characteristics of the comb-drive actuator, several aspects including material type, structural configuration, and actuation characteristic need to be considered. Here, the material types for constructing the heterogeneous structure are typically selected as Si layer and glass substrate, which are anodically bonded together. The different coefficients of thermal expansion between Si and glass possibly affect the conformality of the comb-drive actuator at low temperatures, mainly acting on the flexible structure and further leading to abnormal motion characteristics. The structural configuration of the comb-drive actuator conventionally consists of interdigital comb-finger electrodes and two symmetrically distributed support springs. The stiffness of the support springs is easily affected by the deformation of structural configuration at low temperatures, due to the mismatched thermal expansion coefficients in the heterogeneous structure. Moreover, the equation of the actuation displacement *d* of the comb-drive actuator can be expressed as:

(1) $$d=\frac{F}{K}=\frac{N\varepsilon tV^{2}}{Kg},$$

where *F* denotes the electrostatic actuation force, *K* denotes the spring constant of the support spring, *N* is the number of the comb-finger pairs, *ε* is the permittivity of air, *g* is the gap spacing, and *V* is the actuation voltage. It can be seen that the parameter of the spring constant of the support spring is the dominant factor, which is easily affected by the structural deformation at low temperatures. Therefore, in addition to the conventional structural configuration, the support spring with different spring constants are mainly verified in the following experiments.

Here, the structural parameters of the comb-drive actuator, including the comb fingers, support spring, and displacement sensor, are presented in Table 1. The gap spacing between adjacent fingers, as the essential parameter, is designed as 10 μm by considering the aspect ratio (20:1) for fabricating a vertical sidewall using deep reactive iron etching (RIE). Ten rows of combs are arranged for achieving a large displacement at a relatively low actuation voltage. The initial capacity of the displacement sensor can be calculated by the initial overlap of comb fingers, finger thickness, gap spacing, and number of finger pairs. As the support spring supporting the movable comb is the dominant element in the comb-drive actuator for generating the desired motion, two types of support springs with different spring constants, i.e., 18 (small) and 33 (large) N/m, are adopted for adequately characterizing the influence of different stiffnesses of support springs on the motion characteristics at low temperatures. Here, the lever widths of the two types of support springs are designed as 25 and 30 μm, respectively. On the basis of the structural parameters, an electrostatic force of 3.2 × 10^–3^ N and the corresponding displacements of 89 and 49 μm can be obtained at an applied actuation voltage of 100 V.

Note that *w*/*W*, *l*/*L*, and *t*/*T* represent width, length, and thickness of comb fingers/support spring, respectively.

**Figure 2:**
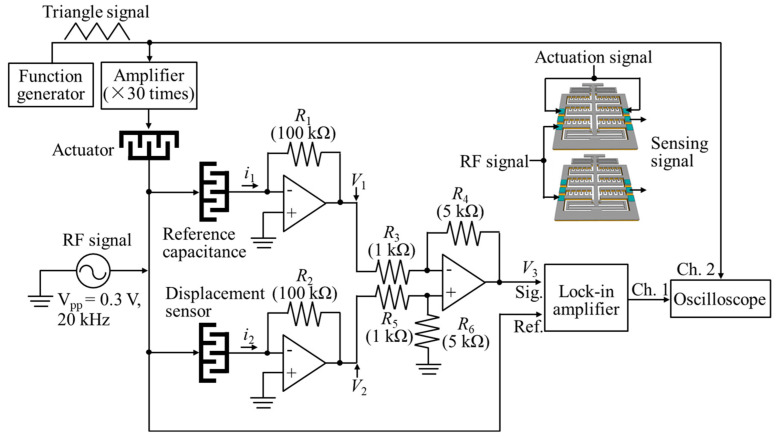
Displacement actuation and sensing circuit of the comb-drive actuator. (The inset shows the circuit interfaces connecting with the comb-drive actuator; the terms “signal”, “reference”, and “channel” are abbreviated as Sig., Ref., and Ch., respectively).

To monitor the motion situation of the comb-drive actuator in the cryogenic chamber, we design a displacement-sensing and signal-output circuit, as illustrated in Figure 2. A triangle wave, provided by a function generator connected to an amplifier (30 times), is applied to the comb-drive actuator to generate a reciprocating translational displacement with time flow. Owing to the motion of the movable comb-finger electrodes, the capacity of the displacement sensor varys synchronously. A sensing circuit, containing two operation amplifiers and a differential amplifier, is utilized to first sense the output displacement signal by converting the current into voltage, and then amplify the voltage signal for easy detection using a lock-in amplifier. Because the designed comb-drive actuator only contains a displacement sensor, we adopt a displacement sensor in another comb-drive actuator as the reference capacitance to realize the differential amplification sensing mechanism. When the reference capacitance and displacement sensor are applied together with a modulation radio frequency (RF) signal of *A*_0_sin*ωt*, the output currents of *i*_1_ and *i*_2_ from each capacitance are presented as:

(2) $$i_{1}=A_{0}\omega C_{1}\cos\omega t,$$

(3) $$i_{2}=A_{0}\omega (C_{2}+dC_{2})\cos\omega t,$$

where *A*_0_, *t*, and *ω* are the amplitude, time, and angular velocity of the RF signal, respectively; *C*_1_ is the capacity of the reference capacitance; and *C*_2_ and *dC*_2_ are the initial capacity and changing value of the displacement sensor, respectively. According to the current-to-voltage conversion, the voltages of *V*_1_ and *V*_2_ from two operational amplifiers are derived as:

(4) $$V_{1}=A_{0}\omega R_{1}C_{1}\cos\omega t,$$

(5) $$V_{2}=A_{0}\omega R_{2}(C_{2}+dC_{2})\cos\omega t.$$

To obtain a high signal-to-noise ratio, a differential amplification is further performed to generate the amplified voltage of *V*_3_:

(6) $$V_{3}=(\frac{R_{3}+R_{4}}{R_{5}+R_{6}})\frac{R_{6}}{R_{3}}V_{2}-\frac{R_{4}}{R_{3}}V_{1}.$$

Moreover, a lock-in amplifier is used to filter the amplified voltage *V*_3_, which is monitored by a digital oscilloscope. Here, two channels, i.e., Channel 1 for displacement signal and Channel 2 for actuation voltage signal, are displayed synchronously in the digital oscilloscope. Finally, we can monitor the actuation performance of the comb-drive actuator in real time, by observing the output displacement signal. Here, the initial capacities *C*_1_ and *C*_2_ of the reference capacitance and displacement sensor are designed as the same value of 4.8 × 10^−13^ F. As the movable comb-finger electrodes are actuated with a maximum displacement of 60 μm, the capacity *C*_2_ of the displacement sensor varies from 4.8 × 10^−13^ to 2.4 × 10^−12^ F. It should be noted that the real resistances of the resistors of *R*_1_~*R*_6_ in the sensing circuit have already been listed in Figure 2. The other parameters including the modulation RF signal and lock-in amplifier are introduced in the experimental section. In addition, a feed-back control system can be further constructed for realizing precious positioning based on the related control algorithms [35,36].

## 3. Fabrication

**Figure 3:**
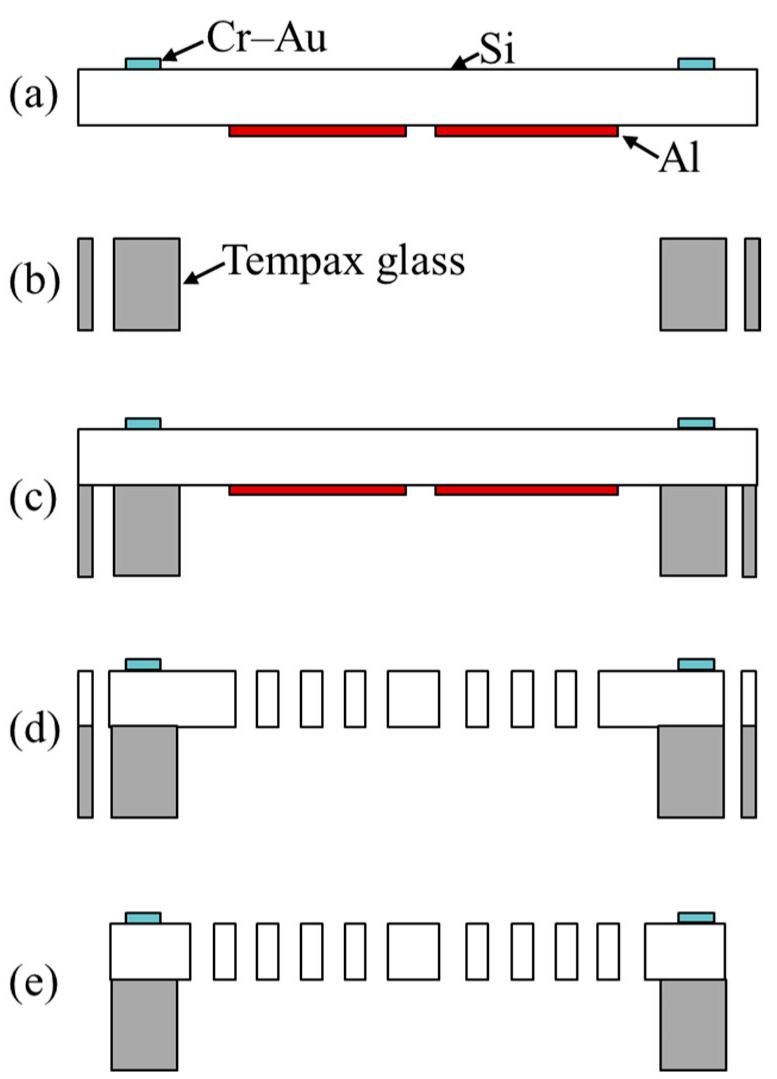
Microfabrication process of the comb-drive actuator based on the heterogeneous substrate of Si anodically bonded onto glass: (**a**) sputter metal layers and lift-off; (**b**) sandblast etching of Tempax glass; (**c**) anodic bonding of Si with glass; (**d**) deep RIE of Si and wet etching of Al; (**e**) YAG-laser cutting of support beams and supercritical drier.

Figure 3 depicts the microfabrication process of the comb-drive actuator from a heterogeneous substrate. Firstly, Si wafer with a thickness of 200 μm and a size of 20 × 20 mm^2^ is used as our device layer for fabricating the functional structures. A thin film of Cr–Au (30–300 nm), as the electrode pads for the comb-drive actuator and displacement sensor, is deposited on the Si wafer and patterned through the lift-off process (Figure 3a). To avoid lateral etching of the functional structures including the comb fingers and support springs during the deep RIE process (Figure 3d), a thin film of aluminum (Al, 300 nm) is additionally sputtered and patterned on the backside of the Si wafer (Figure 3a). Tempax glass with a thickness of 300 μm and a size of 20 × 20 mm^2^ is etched with partially penetrated structures through sandblast etching (Figure 3b). After precisely aligning the Si wafer with the Tempax glass, a heterogeneous structure with a dimension of 20 × 20 mm^2^ is established by anodic bonding at a temperature of 450 °C with an applied voltage of 600 V for 12 min (Figure 3c). Here, the movable comb parts in the Si wafer matches well with the hollowed parts in the Tempax glass. After transferring the photoresist-based pattern into the Si wafer through deep RIE to achieve the desired functional structures, the remaining Al film on the backside of the Si wafer is removed using a wet-etching method (Figure 3d). Finally, the support beams in the Si wafer are cut using a YAG laser to release the movable structures, followed with a supercritical drying of the released structures to reduce the liquid surface tension (Figure 3e).

**Figure 4:**
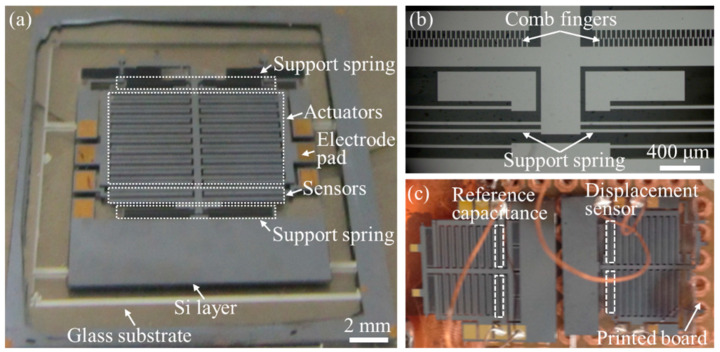
(**a**) Optical image of the fabricated comb-drive actuator (large spring constant type) with the heterogeneous structure of Si anodically bonded onto glass; (**b**) locally enlarged view of the comb fingers and support spring; (**c**) two comb-drive actuators wire-bonded onto a printed board for loading into the cryostat.

Finally, the comb-drive actuators with the heterogeneous structure of silicon anodically bonded onto glass were fabricated based on the aforementioned microfabrication technology. Owing to the identical structural configuration, Figure 4a only presents the optical image of the whole structural morphology of the fabricated comb-drive actuator with large spring constant. The partial structures including the comb fingers and support spring are locally enlarged in Figure 4b, indicating that the functional structures are well patterned for realizing the actuation performance. It should be noted that the upper sides of the fabricated comb-drive actuator, additionally containing the structures for other applications, are not depicted in the schematic diagram of Figure 1b. Figure 4c shows two comb-drive actuators wire-bonded onto a printed board, where the displacement sensor in the left comb-drive actuator acts as the reference capacitance to construct the differential amplification sensing circuit. Thereafter, we load the wire-bonded comb-drive actuators into the vacuum chamber of the cryostat for verifying the actuation performance at low temperatures, as shown in the next section.

## 4. Experiment

**Figure 5:**
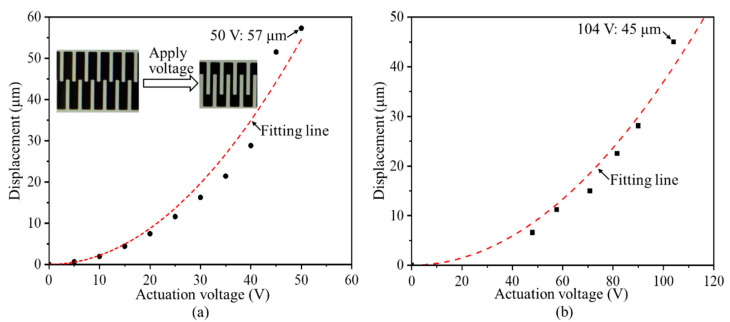
Optical measurement results of the displacements versus actuation voltages of the comb-drive actuators: (**a**) With small spring constant; (**b**) with large spring constant of support springs. (The inset shows the captured motions of the comb fingers at different actuation voltages using a digital microscope.).

Before installing the comb-drive actuators into a cryostat for the low-temperature measurements, the measured displacements from the displacement sensor are calibrated at room temperature using an optical microscope connecting with a digital camera (VHX 100, KEYENCE Corporation, Osaka, Japan). A series of actuation voltages are applied to the comb-drive actuators using a DC power supply, and the overlapping positions of the comb fingers at each actuation voltage are captured for further calculating the actuated displacements, as shown in the insets in Figure 5a. The relationships of the displacements versus the actuation voltages of two comb-drive actuators at room temperature of 298 K are plotted in Figure 5. It can be seen that two types of comb-drive actuators with small and large spring constant support springs provide the maximum displacements of 57 and 45 μm at actuation voltages of 50 and 104 V, respectively. With respect to the different spring constants used in the comb-drive actuators, these measurement results present a good difference in the actuation performances. As compared with the simulation results, the actually actuated displacements of the comb-drive actuators become a little deviated, possibly resulting from the dimensional deviations of the microstructures, such as the levers of the support springs and the gap spacing between the comb fingers. The fitting lines based on the measured data exhibit a good square relationship between the displacements and actuation voltages, verifying the excellent actuation performances of the fabricated comb-drive actuators at room temperature.

**Figure 6:**
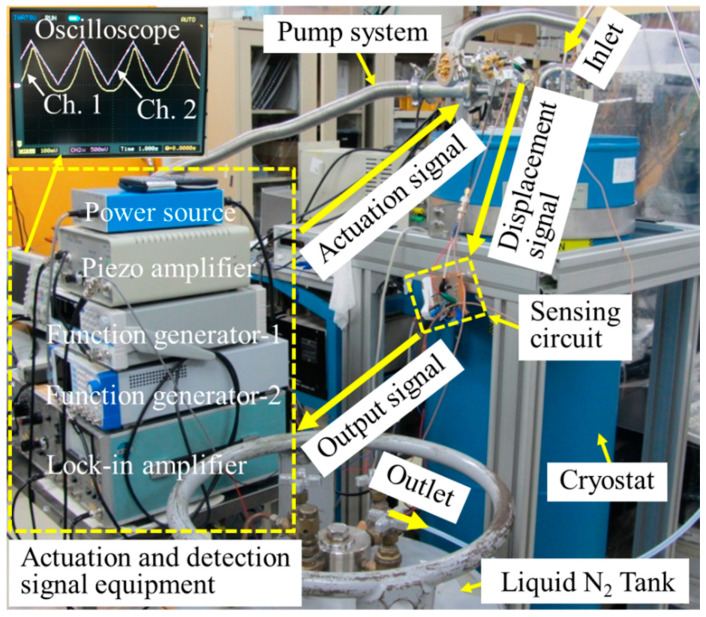
Experimental setup of the cryogenic measurement of the comb-drive actuators fabricated from a heterogeneous structure of Si bonded onto glass. (The inset shows the captured screen from the oscilloscope.).

To investigate the motion characteristics of the fabricated comb-drive actuators at low temperatures, the comb-drive actuators which are wire-bonded onto a printed board are installed into the vacuum chamber of the cryostat, as shown in Figure 6. A pump system, including a mechanical pump and a molecular pump, pumps the vacuum chamber to 4 × 10^−3^ Pa. Thereafter, a liquid nitrogen (N_2_) tank exports the liquid N_2_ into the cryostat to cool down the vacuum chamber. The temperature of the comb-drive actuator is monitored using a temperature sensor in real time. The function generator-1, connected with a piezo amplifier (30 times), offers a triangle actuation signal to the comb-drive actuators. The function generator-2 provides a modulation RF signal (V_pp_ = 0.3 V, 20 kHz) to the displacement sensor and reference capacitance. The power source outputs a stable DC voltage to the operation amplifiers in the sensing circuit. The displacement and reference signals from the comb-drive actuators are sensed and amplified by the sensing circuit. The lock-in amplifier (sensitivity of 5 mV and time constant of 30 ms) detects the output signals from the sensing circuit. Finally, the detected output signals as well as the actuation signals are monitored simultaneously by the digital oscilloscope. The inset in Figure 6 shows the captured screen from the oscilloscope, where Ch. 1 and Ch. 2 represent the displacement signal and actuation-voltage signal, respectively. The measurement results exhibit a good consistency relationship between the actuation voltages and generated displacements at room temperature. Therefore, the established experimental setup can effectively monitor the actuation performance of the comb-drive actuators at low temperatures.

**Figure 7:**
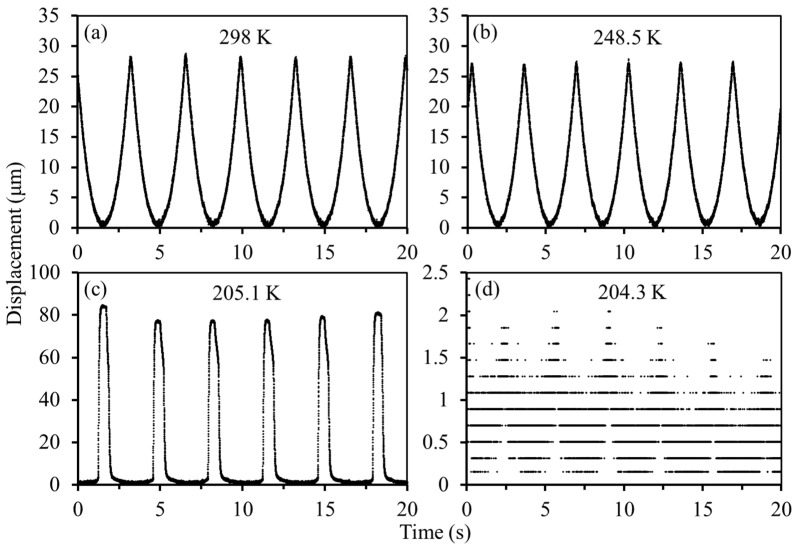
Recorded displacements of the comb-drive actuator with small spring constant at different temperatures: (**a**) 298 K; (**b**) 248.5 K; (**c**) 205.1 K; (**d**) 204.3 K.

When importing liquid N_2_ into the cryostat, two comb-drive actuators with small and large spring constants are cooled down from room temperature of 298 K, respectively. Figure 7 shows the recorded displacements of the comb-drive actuator with small spring constant at different temperatures, where the actuation voltages with a triangle wave are applied. First at room temperature, the detected displacements exhibit a good response to the actuation voltages, and a large displacement of ~28 μm can be achieved with an applied voltage of ~40 V (Figure 7a). With the temperature down to 248.5 K (Figure 7b), the comb-drive actuator still provides good actuation performance. It is worth noting that the actuated displacements at 248.5 K become a little degraded because of the increased spring constant at low temperatures. However, the output displacements become irregular when the temperature is continuously lowered to 205.1 K (Figure 7c). In particular, the comb-drive actuator only responds to the high-level actuation voltages, and the output displacements with a peak waveform are enlarged to ~80 μm. Thereafter, the output displacements suddenly turn to be as small as ~2 μm with a noise level at a temperature of 204.3 K (Figure 7d). From the measurement results in Figure 7c,d, it can be seen that the motion of the movable comb fingers is irregular, resulting in irregular capacity of the displacement sensor. Since the movable comb fingers are supported by support springs, the irregular motion can contribute to the deformation of the flexible support springs at low temperatures, further affecting the spring constants. Therefore, compliance of the deformed support springs at low temperatures cannot comply with the original motion law at room temperature, thereby resulting in irregular motion. Notably, although the experimental temperature difference in Figure 7c,d is only 0.8 K, the generated irregular motions present are different. Because the deformed support springs in this low-temperature range cannot provide compliant movement along the direction of motion, i.e., exceeding the compliance threshold, the irregular motion exited by a certain actuation force possibly exhibits inconsistency.

**Figure 8:**
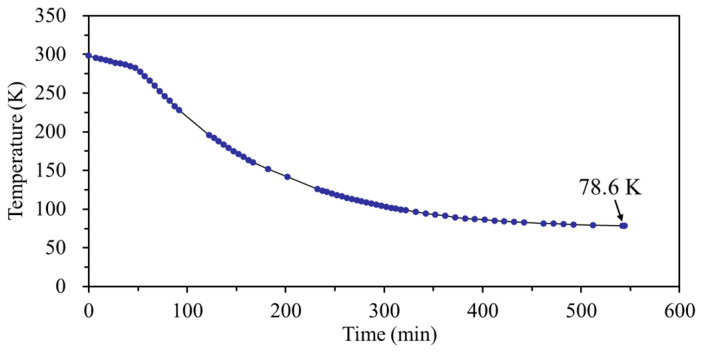
Temperature dropping process of the comb-drive actuator in the cryostat versus time flow.

**Figure 9:**
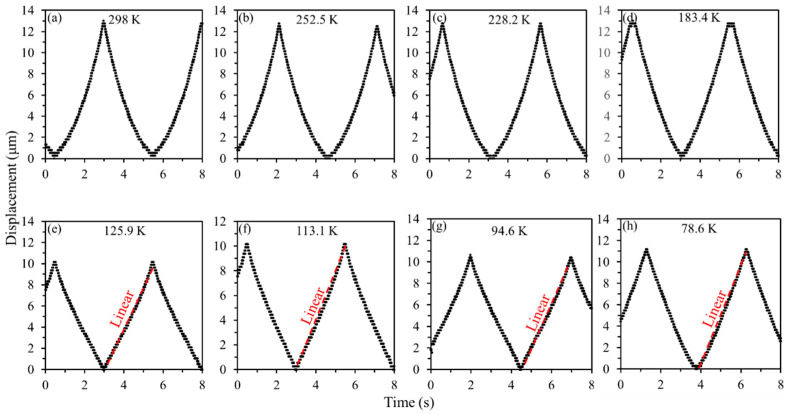
Displacement comparison of the comb-drive actuator with large spring constant at different temperatures: (**a**) 298 K; (**b**) 252.5 K; (**c**) 228.2 K; (**d**) 183.4 K; (**e**) 125.9 K; (**f**) 113.1 K; (**g**) 94.6 K; (**h**) 78.6 K.

Moreover, we also performed another measurement of the comb-drive actuator with large spring constant, to verify the feasibility of the support springs with different stiffnesses on the actuation performance at low temperatures. Figure 8 records the temperature dropping process of the comb-drive actuator in the cryostat versus time flow, where the lowest temperature of 78.6 K, i.e., near the boiling point of liquid N_2_, was achieved after continuously importing liquid N_2_ for ~9 h. The actuated displacements from the displacement sensor at room and low temperatures are compared to visually analyze the actuation performances of the heterogeneous structure-based comb-drive actuator, as illustrated in Figure 9. First, the detected displacements at room temperature of 298 K show a good response to the actuation voltages, and a large displacement of ~13 μm can be achieved with an applied voltage of ~60 V (Figure 9a). As compared with the actuation displacements at room temperature, the obtained displacements at low temperatures in Figure 9b–h become smaller. This can be attributed to the larger Young’s modulus of Si material with decreased temperatures [37], further leading to a larger spring constant and a smaller displacement. However, the obtained maximum displacements, shown in Figure 9e–h, are fluctuating, i.e., not a linear decrease. In addition, the output displacements from the displacement sensor seem irregular as compared with those from room temperature. In other words, the relationship between the displacements and actuation voltages is linear, i.e., not originally square. The abnormal motion characteristics can be ascribed to the deformation of the flexible support springs at low temperatures, further affecting the spring constants. As compared with the measurement results of the comb-drive actuator with small spring constant in Figure 7, it can be seen that the support spring with large stiffness, is capable of withstanding a high strain force, and is more suitable for lower temperature applications. Owing to this spring constant, the safety threshold for achieving a normal actuation relationship can be considered to be a temperature of ~183.4 K, as shown in Figure 9d, whereas the safety threshold just for actuation displacement can reach a temperature as low as 78.6 K, as shown in Figure 9h.

**Figure 10:**
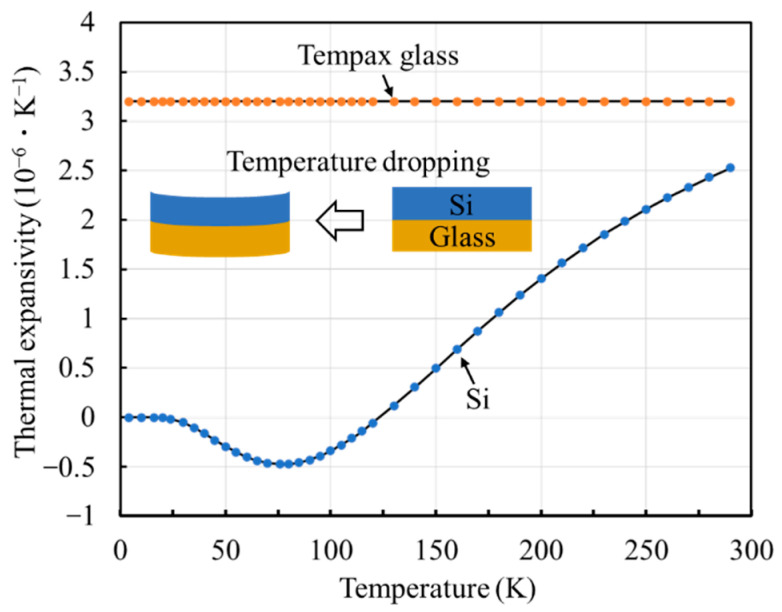
Comparison of thermal expansion coefficients between Si and Tempax glass. (Inset shows the deformation of the heterogeneous structure of Si bonded onto glass with decreased temperature.).

A qualitative analysis of the reasons for the irregular motion of the comb-drive actuator at low temperatures, shows that it can be contributed to the heterogeneous structure of Si bonded onto glass as the fabrication substrate material. Figure 9 plots the changing trends of the thermal expansion coefficients of Si and Tempax glass with respect to the temperatures. It can be seen that the dependence of the thermal expansion coefficient of Tempax glass on temperature is constant, whereas that of the Si material varys with changes in temperature [38]. Owing to the unidentical thermal expansion coefficients, the deformation of the heterogeneous structure of Si bonded onto glass continuously cumulates with a decrease in the temperatures, as shown in the inset in Figure 10. The cumulated deformation easily acts on the support springs and affects the spring constant. To a certain extent, the stiffness of the support springs cannot afford the movable comb fingers at the initial position, resulting in mismatched interdigital comb fingers, i.e., unidentical gaps between adjacent comb fingers. This easily results in a pull-in effect (lateral instability) between the comb fingers, even at a small actuation voltage range [34]. The actuation performance of the comb-drive actuator at low temperatures can be improved by enhancing the stiffness of the support springs to constrain the deformability. It should be pointed out that other possible factors, such as the thickness and structural morphology of the heterogeneous structure, also affect the actuation performances at low temperatures. In conclusion, the heterogeneous structure with different thermal expansion coefficients, such as Si bonded onto glass, is not an ideal material substrate for fabricating a comb-drive actuator with cryogenic applications. It also should be noted that this study only focuses on the experimental demonstration, and does not establish a mathematical model for quantitatively analyzing the functional relationships among the interfering factors. The safety threshold of the heterogeneous-structure-based comb-drive actuators has not been precisely pointed out at low temperatures.

## 5. Conclusions

In this study, we experimentally demonstrated the feasibility of fabricating a comb-drive actuator using heterogeneous structure for cryogenic applications. Comb-drive actuators were fabricated from conventionally heterogeneous structure, where a Si-based device layer was anodically bonded onto a glass substrate. Two kinds of support springs were designed in the comb-drive actuators for verifying the influence of different stiffnesses of support springs on the motion characteristics at low temperatures. The reference capacitance and displacement sensor, also with comb-finger configuration, were embedded in the comb-drive actuator to synchronously monitor the actuation performance at low temperatures. From the experimental results, the motion characteristics at certain low temperatures are irregular, which can be ascribed to the different thermal expansion coefficients of the two types of materials in the heterogeneous structure. This cumulated deformation at low temperatures easily acts on the support springs and further affects the stiffnesses of the support springs. The actuation performance can be improved by enhancing the stiffness of the support springs to constrain the deformability at lower temperatures. Based on this structural configuration, the comb-drive actuator with small spring constant exhibits the possible safety threshold at temperatures >205.1 K, whereas the safety threshold with large spring constant can reach a temperature as low as 78.6 K (a normal actuation relationship at ~183.4 K). Notably, the safety threshold at low temperatures also possibly depends on the thickness, structural morphology, and material type of the heterogeneous structure. It is revealed that the heterogeneous structure with different thermal expansion coefficients is not suitable for fabricating a comb-drive actuator for cryogenic applications. In future study, the SOI wafer could be the replaceable material due to its homogeneous structure, in which a thin SiO_2_ layer on a nanometer order is sandwiched by two Si layers. In addition, a mathematical model needs to be established for quantitatively analyzing the interfering factors on the motion characteristics of a comb-drive actuator at low temperatures.

## Figures and Tables

**Table 1:** Design parameters of the comb-drive actuator.

**Comb Fingers**		
*w* = 15 μm	*l* = 120 μm	*t* = 200 μm
Number of pairs per comb		90 × 2
Rows of combs		10
Gap spacing (*g*)		10 μm
Initial overlap		15 μm
**Support springs**		
*W* = 20/30 μm	*L* = 3500 μm	*T* = 200 μm
Spring constant		18/33 N/m
Number of springs		2
**Displacement sensor**		
Initial capacity		4.8 × 10^–13^ F

Note that *w*/*W*, *l*/*L*, and *t*/*T* represent width, length, and thickness of comb fingers/support spring, respectively.

## Data Availability

Not applicable.
